# Differential impacts of health systems and sociocultural environment on vulnerable populations during the COVID-19 pandemic: lessons from four Asia-Pacific countries

**DOI:** 10.1186/s12889-024-18949-1

**Published:** 2024-06-05

**Authors:** Jakyung Lee, Susan Park, Soong-nang Jang, Katherine Ann Reyes, Fernando Garcia, Carmelita Canila, Joseph Oraño, Alfredo Jose Ballesteros, Tri Muhartini, Sandra Frans, Tiara Marthias, Likke Prawidya Putri, Yodi Mahendradhata, Chuan De Foo

**Affiliations:** 1 Institute for Community Care and Health Equity, Chung-Ang University, Seoul, Republic of Korea; 2 Hyo-tree nursing home, Incheon, Republic of Korea; 3 Seoul National University, Seoul, Republic of Korea; 4 Red Cross College of Nursing, Chung-Ang University, 84 Heukseok-ro, Dongjak-gu, Seoul, 06974 Republic of Korea; 5 Alliance for Improving Health Outcomes, Quezon City, Philippines; 6 College of Public Health, University of the Philippines Manila, Manila, Philippines; 7 Faculty of Medicine, Public Health and Nursing, Universitas Gadjah Mada, Depok, Indonesia; 8 NUS Saw Swee Hock School of Public Health and National University Health System, Singapore, Singapore; 9 Duke NUS Graduate Medical School, Singapore, Singapore

**Keywords:** Health equity, Vulnerable population, COVID-19, Health policy

## Abstract

**Background:**

This study aims to evaluate healthcare systems and pandemic responses in relation to marginalized and vulnerable groups, identify populations requiring urgent care, and assess the differential impacts on their health during the pandemic.

**Methods:**

Data were collected by the Asia-Pacific Observatory on Health Systems and Policies (APO)–National University of Singapore and APO–International Health Policy Program consortium members: Korea, Indonesia, Philippines, and Singapore. Data were collected through a combination of semi-structured interviews, policy document reviews, and analysis of secondary data.

**Results:**

Our findings reveal that the pandemic exacerbated existing health disparities, particularly affecting older adults, women, and children. Additionally, the study identified LGBTI individuals, healthcare workers, slum dwellers, and migrant workers as groups that faced particularly severe challenges during the pandemic. LGBTI individuals encountered heightened discrimination and limited access to health services tailored to their needs. Healthcare workers suffered from immense stress and risk due to prolonged exposure to the virus and critical working conditions. Slum dwellers struggled with healthcare access and social distancing due to high population density and inadequate sanitation. Migrant workers were particularly hard hit by high risks of virus transmission and stringent, often discriminatory, isolation measures that compounded their vulnerability. The study highlights the variation in the extent and nature of vulnerabilities, which were influenced by each country’s specific social environment and healthcare infrastructure. It was observed that public health interventions often lacked the specificity required to effectively address the needs of all vulnerable groups, suggesting a gap in policy and implementation.

**Conclusions:**

The study underscores that vulnerabilities vary greatly depending on the social environment and context of each country, affecting the degree and types of vulnerable groups. It is critical that measures to ensure universal health coverage and equal accessibility to healthcare are specifically designed to address the needs of the most vulnerable. Despite commonalities among groups across different societies, these interventions must be adapted to reflect the unique characteristics of each group within their specific social contexts to effectively mitigate the impact of health disparities.

**Supplementary Information:**

The online version contains supplementary material available at 10.1186/s12889-024-18949-1.

## Background

The 2019 coronavirus disease (COVID-19) pandemic exacerbated pre-existing inequalities in social, economic, and health systems, amplifying the harmful effects of COVID-19 [1–3]. Although researchers generally agreed on who were considered vulnerable to the COVID-19 threat, different interpretations emerged across different national contexts. These vulnerable groups’ health needs are complex and closely related to their socioeconomic conditions. Each country’s healthcare system and social resilience may impact vulnerability characteristics [4] and the outcome of the COVID-19 pandemic [5, 6]. Hence, we must redefine at-risk populations in the context of COVID-19 threats based on the healthcare system of each country and the socioeconomic situation in preparation for emerging infectious diseases [7].

Assessing the healthcare systems and social resilience of different countries may provide insight into the development of effective policies to better respond to public health emergencies. For example, a review of COVID-19 responses in 28 countries suggested that highly effective countries activated comprehensive responses, adapted the health system’s capacity, and preserved functions and resources for both COVID-19-related and non-COVID-19-related care, thus reducing vulnerability in health and well-being [8]. Another study comparing nine high-income countries in the Asia-Pacific region (e.g., Singapore and South Korea) and Europe (e.g., Germany and the UK) showed that interventions in Asian countries were implemented with higher speed, scale, and intensity, leading to early success in the control of COVID-19 [9]. Han et al. [9] suggested that Asian countries’ previous experiences with epidemics, such as Severe Acute Respiratory Syndrome and Middle East Respiratory Syndrome, contributed to high investment in public health infrastructure and public compliance with government restrictions and preventive measures.

However, research on COVID-19 responses in the Asia-Pacific region has been limited. Moreover, comparative studies of COVID-19 responses that included Asia-Pacific countries focused on disease control measures rather than the impact of such policies on vulnerable populations [10, 11]. Southeast Asian countries faced severe challenges to COVID-19, but their unique interventions, such as the use of community health volunteers, may provide insights for other countries [12]. Asia-Pacific countries share several cultural and environmental features: livestock and poultry rearing methods, high population density, high population migration rates, and changes in their ecological environment due to climate change. However, cooperation in health security among Asia-Pacific countries is stalling. Therefore, comparing and considering the policy responses in Asia-Pacific countries is necessary to improve the response levels to the epidemic and promote health justice through international cooperation.

We chose four Asia-Pacific countries that are members of the Asia-Pacific Observatory on Health Systems and Policies (APO)–National University of Singapore (NUS) and APO–International Health Policy Program (IHPP) consortium for this study: South Korea, Indonesia, the Philippines, and Singapore. The countries varied in the levels of epidemiological outcomes, including confirmed cases and death rates, as well as in the strategies to respond to the pandemic. The country selection is also based on the differences in healthcare systems, and the selected countries represent a range of income levels. We assumed that the differences in socioeconomic and environmental factors would affect COVID-19 response policies and the health outcomes of vulnerable populations in the four countries. We investigated the characterization of marginalized and vulnerable populations in the four countries and collected country-specific data, such as specific healthcare policies, and their applications.

The goals of this study are to (1) evaluate the healthcare systems and COVID-19 responses focused on marginalized and vulnerable populations during the pandemic; (2) identify the marginalized and vulnerable populations who required urgent care during the COVID-19 pandemic; and (3) assess the differential impact of the COVID-19 pandemic on health and well-being in vulnerable populations. We aim to provide new perspectives on a country’s pandemic preparedness while also highlighting effective management methods for improving response plans for other emerging infectious diseases.

## Methods

Data were collected from the APO–NUS and APO–IHPP consortium members from Korea, Indonesia, the Philippines, and Singapore, over the course of February 2022. This study triangulates qualitative methods, including semi-structured interviews, discussions with relevant experts through workshops, and reviews of national policy documents. The authors from each country determined the vulnerable and marginalized groups within their nations and complete semi-structured interview questionnaires regarding national responses to these groups. The interview items were based on the Global Health Security (GHS) index and the Joint External Evaluation (JEE) tool, both validated by previous studies [13, 14]. Table 1 lists interview items and the full interview questionnaires is available in Supplementary Material 1. To complete the questionnaires, the authors for each country analyzed policy documents and secondary data, and sought expert opinions to complement the collected data. Relevant experts from each country included members of international organizations, university professors specialized in related fields, and government officials from departments dedicated to infectious disease response. Subsequently, discussions were held among the authors from each country to reach a consensus.

The data from each country were integrated by the Korean research team in March 2023. Two researchers separately analyzed initially, then they cross-examined each other’s analysis. The part of results which were the same interpretations by two researchers were accepted, but when the interpretations differed, it went through a process of reaching agreement though discussion. All content analysis processes and results were reviewed for agreement by another researcher who are PhD in Public Health. Finally, all participating researchers reviewed the analysis results and reached an agreement.

**Table 1:** Overview of the healthcare system and social resilience measures for the study

Category
Prevention of the emergence or release of pathogens
Early detection and reporting of epidemics of potential international concern
Strength and quality of laboratory systems
Accessibility and transparency of surveillance data
Case-based investigation
Rapid response to and mitigation of the spread of an epidemic
Emergency preparedness and response planning
Risk communication
Access to communications infrastructure
Sufficient and robust health sector to treat the sick and protect health workers
Health capacity in clinics, hospitals, and community care centers
Healthcare access
Commitments to improving national capacity, financing, and adherence to norms
Overall risk environment and country vulnerability to biological threats
Socioeconomic resilience
Public health vulnerabilities

JEE = Joint External Evaluation Tool; GHS = Global Health Security

## Results

Table 2 summarizes the priorities of vulnerable populations in four countries, as determined by policy documents, secondary data, and expert opinions.

**Table 2:** The vulnerable populations requiring specific attention based on the country’s experience during the COVID-19 pandemic

	Korea	Indonesia	Philippines	Singapore
1st priority	Older people	Healthcare workers, assistants for healthcare workers and supporting staff working in Health Service Facilities.	Older people	Older people/Seniors
2nd priority	Women	Older people, market traders, Religious leaders, Government employees, police and military, journalists, tourism workers, civil servants (firefighter), athletes and public transportation workers (formal and informal).	Women	Migrant workers
3rd priority	Children and Youth	Located in remote areas, the poor, people with disabilities and HIV sufferers.	Adolescents, children, and youth	Women and pregnant women
Not listed above	LGBTI and homeless people	Pregnant women, children (6–17 years) and residents who do not yet have an ID or residence number.	Slum dwellers, people in informal settlements, and homeless persons; People in extreme poverty or facing insecure and informal work and incomes.	Students (from preschool to secondary school)

### Common vulnerable populations in four countries

#### Older adults

As a common vulnerable group in the four countries, older adults were considered a priority target group. The older population (generally 60 years and above) is deemed a vulnerable group for many reasons. First, they are a population more predisposed to severe conditions due to pre-existing medical conditions. Due to the restricted access to healthcare services and chronic disease diagnosis services during the pandemic, this population may suffer from further complications and deterioration from pre-existing comorbidities. Comorbidities also predispose older people to more severe symptoms and outcomes if they are infected with the virus [15].

Furthermore, cluster infections have focused on long-term care facilities, resulting in more deaths due to the health vulnerabilities of the subjects [16]. Nursing home residents were reported to comprise 25% of the deaths due to COVID-19 in the US and 50% in European countries (e.g., France and Ireland) [17]. Nursing homes and assisted living facilities in many countries have failed to prevent the virus from spreading among older residents and staff timely. In addition to the chronic shortage of care workers, the devaluation of older adults in facilities and their caregivers during the COVID-19 pandemic exacerbated the care crisis in long-term care facilities [18]. To mitigate such damage, measures to restrict visitors to senior care facilities caused additional problems, such as emotional distress or victim abuse.

#### Women

Women have been prioritized as one of the most vulnerable groups during the pandemic, as they bear most of the burden at home (performing both work and caregiving tasks), which is further intensified when work-from-home setting and home-based learning arrangements for their children were implemented during tightened movement restrictions. During the COVID-19 pandemic, many family caregivers, who are women, experienced anxiety, loneliness, and workplace disruptions, such as loss of jobs and wage reduction [19]. Domestic violence against women also increased. In Singapore, the Association of Women for Action and Research reported an exponential increase in calls to its helplines from women since the beginning of 2020.

Furthermore, women’s sexual, reproductive, and maternal health were affected by the disruptions in essential health services. A review of 95 studies showed that pregnant women with COVID-19 were more likely to experience adverse health outcomes, including preeclampsia, preterm birth, maternal mental health issues, and even deaths [20]. There was also a lack of clear and evidence-based guidelines for COVID-19 patients on delivery and breastfeeding, which may potentially harm pregnant women’s health [21]. Pregnant women were reported to be excluded from most COVID-19 treatment and vaccination programs., hindering the development of effective treatments for them [22].

However, there is little (if any) policy specifically addressing women’s heightened needs during this health emergency. Most of the initiatives are created from the ground up and through the lens of civil society. Despite the gendered impact of the COVID-19 pandemic on health, public health research and policy development that consider gender differences are limited [23].

#### Children and youth

Children with mild symptoms may act as the sources of COVID-19 transmission in schools and community settings [24]. More importantly, the health and well-being of the children were negatively affected by school closures and social distancing measures [25]. Young children experienced high levels of stress, anxiety, and disturbance of sleep, especially those with low socioeconomic status or pre-existing mental health issues [26]. The disruption of essential health services affected the health of children and adolescents, particularly those living in vulnerable conditions [25]. Restrictions during the COVID-19 pandemic also decreased physical activity among young people under the age of 18 [27]. These unfavorable conditions for the growth of young children during the COVID-19 pandemic may have a long-term impact on their physical and mental health. Because previous COVID-19 studies primarily focused on adults, more research is needed to understand the long-term health effects of COVID-19 on the younger population and identify ways to mitigate its negative impact on them.

### Other vulnerable populations by country

#### Korea

##### LGBTI people

In May 2020, over 200 infected cases were linked to an outbreak in a nightclub in Seoul’s Itaewon district [28]. A news report that a person with a confirmed infection visited a gay club sparked homophobia [29]. In response, LGBTI people were reluctant to test for COVID-19, revealing the privacy invasion issue in the current testing system. LGBTI organizations advocated for local governments to implement anonymous testing to reduce LGBTI people’s fear of retaliation. A large body of prior research has shown that LGBTI people face significant health inequalities due to heteronormativity, minority stress, victimization, discrimination, and stigma [30]. The case of Itaewon in Korea demonstrated that the LGBTI population’s health inequalities were exacerbated in the country’s response to COVID-19. Health inequality occurs in situations where heterosexuality is the norm [31]. In Korea, conversion therapy that infringes human rights continues, despite international organizations, such as the United Nations, urging to cease the practice [32]. This social environment contributes to high rates of drinking, smoking, depression, and suicidal ideation among LGBTI people in Korea [33–35]. Nevertheless, same-sex couples in Korea do not receive the same spousal health insurance coverage as heterosexual couples [36].

##### Homeless people

Homeless people were a blind spot for Korean national strategies against COVID-19. Due to unclear residences, the health authorities had difficulty conducting epidemiological investigations. Seoul’s government operated temporary shelters, but the lack of adequate space for physical distancing made them vulnerable to infection. Moreover, homeless people experienced various challenges in their daily lives during the COVID-19 pandemic, including employment and the use of social welfare services [37]. Due to the closures of local soup kitchens and healthcare organizations, homeless people on the streets and shelter dwellers were also deprived of free meals and health services during the COVID-19 pandemic [38]. Furthermore, since the government designated public hospitals as specialized facilities for COVID-19 patients, many homeless people had restricted access to medical services.

#### Indonesia

##### Healthcare workers

High infection risks and mortality among healthcare workers have been reported in Indonesia [39]. A significant number of caseloads resulted in a high workload of healthcare workers, which was aggravated by a lack of personal protective equipment (PPE) and medical supplies [39, 40]. During the early stages of the COVID-19 pandemic, the PPE shortage was a commonly mentioned cause of death for healthcare professionals worldwide [41]. Moreover, the number of healthcare workers in Indonesia dealing with the demands of COVID-19 cases was insufficient. As the number of COVID-19 patients in Indonesia increased, so did the workload of medical workers, resulting in long and irregular working hours. These working conditions caused psychological distress for healthcare workers [42]. A lack of staff, uncertainty about COVID-19 control, and inadequate protection measures were identified as key stressors among Indonesian healthcare workers [43].

##### Religious leaders

Although Indonesia is a religious country with many denominations, most of the population is Muslim. Massive religious gatherings in Indonesia aided the spread of the COVID-19 virus. The public saw government gathering restrictions and social distancing measures as threats to religious traditions [44]. Similarly, in South Korea, gatherings of the “Shincheonji” Church were a source of outbreaks in the early phase of the COVID-19 pandemic. These examples highlight the importance of comprehending the effects of religion, culture, and social factors on people’s perceptions and behaviors during pandemics. People of different faiths may react differently when it comes to following preventive health measures.

#### Philippines

##### Slum dwellers and people in informal settlements

With 42,857 people per square kilometer, Manila is the world’s most densely populated city. COVID-19 ripped through high-density slum dwellings in Metro Manila [45]. According to World Bank data, approximately half of the urban population lives in slums [46]. People living in high-density areas are more vulnerable to the negative effects of community quarantine, such as economic difficulties, food insecurity, and domestic violence. Furthermore, in 2020, typhoons Vamco (Rolly) and Goni (Ulysses) wreaked havoc on homes. Consequently, at least 30,000 people in Manila have been displaced and forced to live in communal shelters, making it difficult to follow the recommended social distancing and self-quarantine measures.

#### Singapore

##### Migrant workers

The spotlight was on migrant workers in Singapore, in part due to social factors and the existing policy climate [47]. Most COVID-19 cases in Singapore occurred during the initial wave among migrant workers, following several large outbreaks in migrant worker dormitories [48]. Independent observers and local nongovernmental organizations (NGOs) pointed to deficiencies in workers’ dormitories regarding the provision and quality of basic necessities. As of May 6, 2020, there were 17,758 confirmed cases of COVID-19 among dormitory workers (88% of 20,198 nationally confirmed cases) [48]. Due to their poor living conditions, migrant workers could not effectively practice public health measures, such as social distancing. Migrant workers in Singapore are generally low-skilled, young blue-collar workers from Bangladesh, India, China, Thailand, and Myanmar. During the early stages of the outbreak, the government isolated all dormitories and established medical units of doctors and nurses stationed at the dormitories to care for the workers [49]. Complete movement restrictions increase depression and stress symptoms [50]. Additionally, several migrant workers attempted suicide at their dormitories [51]. The government also prioritized testing for migrant workers, and as of December 2020, 54,505 dormitory residents had tested positive [49]. Although Singapore implemented adequate measures to control COVID-19 in community settings, delayed responses for the migrant worker population led to rapid increases in the number of COVID-19 cases that partially triggered a circuit breaker lockdown early in the pandemic to reduce community transmission [52].

Even before COVID-19, migrant workers were more likely to be employed in low-wage jobs with long working hours and hazardous working conditions [53]. Previous research has shown that immigrants are more likely to be exposed to pesticides and chemicals and often have higher workloads than non-migrant workers [53–55]. However, it is difficult for migrant workers to access adequate healthcare due to inadequate labor protection measures, limited access to health insurance and limited eligibility for legal healthcare [56].

### National response against COVID-19 focused on vulnerable populations

Table 3 shows the national response to COVID-19 and the effectiveness of national policies and implementation for marginalized and vulnerable populations. The authors assessed the effectiveness of health policy and implementation for vulnerable populations.

**Table 3:** The strength of health policy and implementation for marginalized and vulnerable populations

			Rating the strength of health policy and implementation for vulnerable populations							
			**Korea**		**Indonesia**		**Philippines**		**Singapore**	
			Total population ^1)^	Vulnerable population ^2)^	Total population	Vulnerable population	Total population	Vulnerable population	Total population	Vulnerable population
**Average**			4	3.2	3	1.6	3	2.7	3	4.4
**Category**	**Indicator**	**Description**	**Description**	**Score**	**Description**	**Score**	**Description**	**Score**	**Description**	**Score**
Prevention	Testing	Target people	Anyone	4	Disabled women, disabled people	1.5	Older people	3	Migrant workers, older people, children	4.7
	Vaccine	Target people	Healthcare workers, institutionalized people, and older people	4	Older people, informal workers, disabilities	1.5	Older people	3	Healthcare workers, older people, immunocompromised	5
Detection	Surveillance	Ethical considerations during surveillance	No ethical consideration, public disclosure of private information	1	No ethical consideration	1	Protected under the Data Privacy Act	3	Anonymized data, only be used for contact tracing	5
	Case-based investigation	Case investigation and contact tracing, quarantine, isolation	Extensive active case investigation, limited capacity of the public health sector. In the early stage of the pandemic, confirmed cases were isolated in the residential treatment center, but now the principle is self-quarantine at home.	4	No case tracing	1	Contact tracing was conducted using the “demographic vulnerability tool” based on age and housing condition. Community isolation was allowed. There are no provisions specific to vulnerable populations.	3	Tracetogether app for contact tracing and passive and active community surveillance. In spite of isolation and quarantine shifted to a home-based and self-monitoring approach.	5
Response	Emergency preparedness and response planning	If an overarching national public health emergency response plan is in place, does it include considerations for pediatric and/or other vulnerable populations?	National public health emergency response plan; consideration of vulnerable populations: disabilities, older adults, children, and immigrants	3	National disaster management plan 2020–2024; Child protection, especially children who are victims of disasters and refugees	3	Manual operations on health emergency and disaster response management; consideration of vulnerable populations children and older people	3	All patients diagnosed with COVID-19 will be able to receive treatment at any point of care and at any level of care in Singapore.	4
	Risk communication	Does the risk communication plan outline how messages will reach populations and sectors with different communications needs (e.g., different languages, within the country, media reach)?	The government held a press briefing twice a day, and contact tracing data of infected people has been released on the Internet. All social fields disclosed the information transparently. However, the community, especially for marginalized people, is limited in the communication process.	4	The Ministry of Health has provided daily press briefings and regular press releases. Daily media monitoring, management of false information	2	The COVID-19 risk communication toolkits respond to the needs of certain vulnerable segments of the population, with the notable exception of the economically disadvantaged.	3	The taskforce was transparent and frequent in providing situational updates, as well as new guidance and its associated rationale through traditional and social media channels, and regular press conferences.	3.4
	Access to communications infrastructure	Access to a mobile phone or Internet	Variety of government services such as the regular sending of text messages and vaccination reservations using the Internet based on the high penetration rate of the Internet and cellular phone. However, the rate of vulnerable groups is low compared to the total population.	4	Indonesia has a high penetration rate for cellular phones. However, Internet access is limited (71.6%). The Internet penetration rate is not evenly distributed in rural areas.	1	Cellular phone 80.8% in women, 76.7% in men; Cellular coverage is still patchy for certain regions and geographically isolated areas. Internet access is limited (64.1%).	1	The penetration rates of the Internet and cellular phone are high. The government launched the Seniors Go Digital program in May 2020; Migrant workers were allowed to return to work, but had to report their daily temperatures using mobile applications.	2
Health system	Health capacity in clinics, hospitals, and community care centers	Human resources available for the broader healthcare system, capacity of facilities.	In the early stages of the pandemic, it was impossible to secure the number of beds and residential facilities for the treatment and accommodation of confirmed cases. There are regional variations in public hospitals.	3	During the first and second wave of the pandemic, Indonesia experienced a lack of human resources in the hospitals and primary healthcare levels.	3	Policies and programs enacted to strengthen the capacity of the health system, such as deployment programs and the establishment of new facilities.	3	To complement the public health sector while rapidly increasing the capacity of the general health system, private sector providers have been involved since the beginning of the pandemic.	5
	Healthcare access	Access to healthcare, guaranteed paid sick leave	Decreased access to medical care for patients with other chronic diseases	2	None.	1	2019, all Filipinos are eligible for the benefit available for health services under PhilHealth; no specific provisions for the protection of vulnerable segments of the population.	3	Everyone has the right to access to healthcare in Singapore.	5
	Coverage health insurance	Medicare/Medicaid, out of pocket expenses	National health insurance fully covered treatment costs for COVID-19, and the government provides 93 types of financial support related to COVID-19.	4	None.	1		2	COVID-19 related services are provided for free; but unvaccinated will have to pay their own bill if they contract COVID-19.	5

#### Korea

In Korea, large-scale rapid testing was freely available throughout the country. Korea’s reaction to COVID-19, represented by the “3T” approach (testing, tracking, and treatment), was recognized as a successful response early in the pandemic [57]. Tracing involved an epidemiologic investigation of all people who had had contact with confirmed cases for 14 days using various data sources, such as mobile phone location data, CCTV, and credit card usage history. In the early stage of the pandemic, the routes of movement and patients’ personal information were revealed. Although the information disclosed was anonymous, there were concerns that it could be used to identify patients’ identities. As a result, guidelines were revised several times, and the extensive tracking system for confirmed cases was suspended beginning in April 2022. Based on the tracing results of confirmed cases and their close contacts, quarantine was the primary measure to combat COVID-19. In the early stages of the pandemic, confirmed cases were isolated in healthcare facilities. Except for patients with high-risk health conditions, patients were allowed to be isolated at home once the patient population exploded. Vaccination and treatment were top priorities for older adults and those suffering from underlying diseases.

Korea had the highest response capacity to infectious disease threats of the four countries studied based on the GHS score. However, public disclosure of the tracking results represented serious violations of personal privacy and resulted in the social stigma of individuals with confirmed COVID-19 cases. Risk communication strengthened community awareness of COVID-19 status and helps to mitigate economic losses. However, there was a lack of response to protect vulnerable populations, particularly victims of discrimination and violence [58]. People with COVID-19 symptoms may have avoided testing for fear of exposing personal information.

#### Indonesia

During the first and second waves of the pandemic, Indonesia experienced a shortage of human resources in hospitals and primary care settings. Many health workers contracted COVID-19, disrupting the health service. Surveillance in Indonesia was passive due to the weak implementation of contact tracing. The government would bear the costs if the public primary health center and the health office carried out the tracking. On the contrary, if private health facilities conducted the contact tracing, the costs were paid.

Indonesia implemented policies specific to disabled people. These included ensuring easy access to COVID-19 testing, free health services, health protection, psychosocial support, home visit services for public health consultations and those related to COVID-19. It also includes providing relevant information about disabilities to healthcare workers in order for them to understand the specific needs of people with disabilities. However, the instructions were less specific; their content was more in the form of messages and considerations for facilitators and assisting institutions for people with disabilities rather than in the form of concrete action proposals that were easy to implement.

#### Philippines

The government used the “Vulnerable Populations as Identified in the Demographic Vulnerability Tool” to prioritize contact tracing. This tool considers the risk of infection by age and population density of the environment. The Department of Health issued Department Memorandum No. 2020 − 0189, which specified the close contacts prioritized in contact tracing, including “vulnerable populations as identified in the demographic vulnerabilities tool.” The tool was developed based on epidemiological investigation evidence of COVID-19 cases in urban areas like Manila in the early stages of the pandemic. This tool considers demographic vulnerability based on three conditions: (1) more than four household members living in a housing unit 20 m^2^ or smaller; (2) the total number of people 60 years old and over living in a housing unit larger than 20 m^2^; and (3) the total number of people 60 years old and over living alone in a housing unit larger than 10 m^2^.

The government imposed a lockdown policy for Metro Manila, which included school closures, a ban on large gatherings, community quarantine, a series of stay-at-home orders, and movement restrictions in designated areas. Furthermore, the Philippine government provided continuous social and economic support to those affected by the response to the pandemic, such as the Republic Act (RA) 11,469, which provided emergency subsidies to low-income households. Despite economic assistance policies, the amount provided by RA 11,469 was insufficient to meet the needs of families during prolonged periods of lockdown or unemployment.

#### Singapore

Extensive testing was conducted with migrant workers in focus. The Ministry of Manpower (MOM) prioritized PCR testing for migrant workers in essential services to work safely during Singapore’s circuit breaker period. In August 2020, Fast and Easy Testing–Rostered Routine Testing (RRT) was implemented in this population, with each migrant worker receiving a nose swab every 14 days. Unvaccinated workers were required to test on the 3rd, 7th, and 11th days following each RRT date. Once a high proportion of migrant workers had been fully vaccinated, the MOM announced that the RRT criteria would be waived for certain groups of migrant workers, including those who had been fully vaccinated; construction, marine and process sector workers; front-line workers in dormitories and onboarding centers; and migrant workers’ recreation centers. However, unvaccinated workers would still be required to undergo RRT every three days. Through effective social distance measures, Singapore successfully suppressed the number of COVID-19 infections and deaths. Quarantine measures for migrant workers were a major factor that suppressed community transmission. However, physical and emotional sequelae after isolation revealed the vulnerability of migrant workers in Singapore.

## Discussion

This study explored the effects of four Asian-Pacific countries’ national COVID-19 responses and social factors on the most vulnerable population groups. We reviewed the vulnerable groups commonly identified in the four countries: older adults, women, children, and adolescents. The results also identified vulnerable groups in each country’s sociocultural environment. For example, in Korea, LGBTI and homeless people were identified as vulnerable populations in Korea. Meanwhile, healthcare workers in lower-middle-income countries, such as Indonesia, were shown to be extremely vulnerable during the COVID-19 pandemic. Housing vulnerability was identified in high-density cities in the Philippines, whereas in Singapore, high-intensity tests and quarantine policies for migrant workers were found to have unintended consequences.

All reviewed countries implemented vaccination and testing policies that took into account their vulnerable populations, including older adults, women, children, and healthcare workers. Due to restricted access to health services during the pandemic, the health of older people may have deteriorated due to pre-existing comorbidities. Moreover, wide range of women with various situations can be categorized as vulnerable population in each country. Women bear most of the burden at multiple roles including work, house chores and caregiving tasks, which is further intensified when work-from-home setting and home-based learning arrangements for their children were implemented during tightened movement restrictions [19]. Many female family caregivers experienced anxiety, loneliness, and employment disruptions [19]. Increased domestic violence against women and disruptions to essential health services affected women’s sexual, reproductive, and maternal health [20, 21]. The struggle of healthcare workers due to increased workloads and a lack of resources was a problem experienced in both high-income and low- or middle-income countries [59]. Furthermore, a large portion of healthcare workers around the globe are women, which doubled the burden and hazards for them. The disruption of essential health services also adversely affected the health of children and adolescents, especially those living in vulnerable conditions [25].

However, this study revealed insufficient targeted responses for socially vulnerable groups in many countries. The results showed that gaps in health inequality can be exacerbated when decisions are made without considering vulnerable groups in the population. For example, three countries considered vulnerable populations without adequate housing. People in informal settlements often face many challenges that can undermine health equity, ranging from poverty, inadequate infrastructure, and housing insecurity [60, 61]. As a result, slum dwellers are at a higher risk of infectious and noncommunicable diseases, mental health effects, and injuries from violence and traffic accidents [60, 61]. In addition, urban slum dwellers face conditions that significantly impact health inequity, such as spatial, political, and economic exclusion, compared to city dwellers [62].

The implementation of social policies by the government also had unintended consequences. For instance, physical distancing measures, such as stay-at-home policies, school closures, bans on social gatherings, and limited contact for certain populations, may have shown health benefits in slowing COVID-19 transmission. However, it also resulted in limited access to health and social services and social isolation. The significant increase in older adult abuse and adolescent suicide rate [63, 64] during these periods could be attributed to COVID-19 restrictions [65]. The response to COVID-19 revealed limitations in reaching those with low access to the healthcare system. Continuous research is needed to understand the long-term health effects of COVID-19 on vulnerable populations.

Our study showed the importance of equity in health and basic needs in protecting human rights. A comprehensive approach is required to protect vulnerable populations during and after pandemics. Governments must understand that the policies for public health responses may have varying effects on different populations, particularly on the marginalized ones. Therefore, it is essential to consider the various social factors that may mediate the impact of the COVID-19 pandemic. When developing public health strategies, the government must prioritize identifying and protecting vulnerable populations. Clear risk communication and support measures for vulnerable populations should be implemented immediately. More information transparency is needed to minimize public confusion and stigmatization of marginalized community members. When a quarantine is necessary, public health authorities should provide adequate information about protocols and basic supplies to minimize the negative consequences of isolation. Although proactive disease prevention strategies are effective, ethical considerations should also be emphasized. Privacy protection, for example, should be considered during contact tracing. It is also critical for each nation to invest in its public health sector to increase capacity and access to healthcare services and to develop a sustainable healthcare system.

The analysis of the four countries provides important insights for mitigating current challenges and preparing for the post-pandemic era. The COVID-19 response policies towards LGBTI individuals and migrant workers in Korea and Singapore suggest implementing anti-discrimination policies in conjunction with public health strategies and incorporating more detailed human rights considerations. The case of disease transmission among slum dwellers in the Philippines highlights the impact of housing conditions on health, underscoring the importance of improving housing and providing economic support. Additionally, the healthcare crisis in Indonesia due to a lack of medical resources reveals the need for strengthening healthcare infrastructure and supporting healthcare workers. The differences in vulnerable populations across countries offer information on which health issues should be prioritized for improvement in epidemic crisis responses specific to each nation.

Furthermore, our comparative analysis highlights the importance of cooperation between nations to combat the pandemic by illustrating similarities and differences in public health strategies between countries and their impact on vulnerable populations. A previous study reported that despite the initial variations in public health measures’ speediness, strictness, and resourcefulness during the COVID-19 pandemic, the measures became similar in most countries over time [13]. As time passed, the lessons learned from various countries may have provided practical guidelines for controlling the pandemic. Beyond knowledge sharing, international solidarity is critical for recovery. Moreover, global governance and increased collaboration in surveillance, research, and best practices are critical for resilient responses [66]. Such a collaborative approach would aid in identifying effective ways to protect vulnerable populations and improve health equity during the pandemic.

This study had several limitations. First, this study may not capture all policy changes over time due to the rapid changes in policies and support measures during the COVID-19 pandemic. Many policy measures may have changed since the time this article was written. Second, this may lead to biases in rating priority levels due to the subjective nature of the experts’ opinions. This study involved relevant experts who are representative members from each country in international organizations, aiming to reflect the situation of their respective countries accurately. These experts used their social and academic networks to collect advice that would help identify the objective characteristics of each nation. It was anticipated that objective prioritization of issues would be feasible through the involvement of these experts and the interviewees they selected. However, there are inherent limitations to this method, primarily because it relies heavily on the perspectives of selected experts. Third, while detailing the characteristics of the vulnerable populations would aid in targeting policy interventions toward specific groups, this was not done in the current study. Consequently, because the level of vulnerability was not assessed in detail to identify high-risk subgroups, the definition of vulnerable groups relied largely on the opinions of individual researchers, potentially limiting the applicability of the findings to broader or different contexts. Fourth, this study is grounded within interpretative approach; thus, we did not gather demographic information of interviewees because the study did not only use data from interviews but also multiple resources for each item.

## Conclusions

The COVID-19 pandemic challenged health systems worldwide. Understanding the impact of healthcare systems and social resilience on vulnerable populations during the COVID-19 pandemic is critical. Our findings revealed that COVID-19 exacerbated existing inequalities in social conditions, gender, and healthcare. Across the four countries, vulnerable groups were consistently identified as older adults, women, children, and adolescents. Meanwhile, LGBTI people, healthcare workers, slum dwellers, and migrant workers were also highlighted as highly vulnerable. Depending on the social environment and context of a country, the degree and types of vulnerable groups might be various in each country.

The results suggest the importance of emphasizing equity in healthcare and human rights protection to mitigate the pandemic’s negative impact. Most importantly, measures to ensure universal health coverage and equal accessibility to health care must be specified based on the most vulnerable groups even though there are the common and unique features among vulnerable groups in each society.

### Electronic supplementary material

Below is the link to the electronic supplementary material.

Supplementary Material 1

## Data Availability

Data will be made available from the corresponding author, SJ, on reasonable request.
