# Supplementary material for: Differential impacts of health systems and sociocultural environment on vulnerable populations during the COVID-19 pandemic: lessons from four Asia-Pacific countries
Source: BMC Public Health. 2024 Jun 5;24:1501. doi: 10.1186/s12889-024-18949-1 (PMC11151645; doi:10.1186/s12889-024-18949-1)
Supplement: Supplementary file 1 — Supplementary Material 1 [file 12889_2024_18949_MOESM1_ESM.docx]

**Supplementary Material: A forms of expert opinion survey**

| **Participating country** |  |
| --- | --- |
| **Co-authors** |  |

1. Please select the marginalized and vulnerable populations who require specific attention based on your country’s experience during the COVID-19 pandemic.

| 1. Older people 2. Women 3. Adolescent, children and youth 4. People with disabilities or mental health conditions 5. Migrants, refugees, stateless and internally displaced persons, conflict-affected populations 6. People in detention or in institutionalized settings (e.g. persons in psychiatric care, drug rehabilitation centers, old age homes) 7. Slum dwellers, people in informal settlements, homeless persons 8. Small farmers, fishers, pastoralists, rural workers in informal and formal markets, and other people living in remote rural areas as well as urban informal sector and self- employed who depend on market for food 9. The food insecure, particularly in countries affected by prolonged conflict and crisis 10. People in extreme poverty or facing insecure and informal work and incomes 11. Groups that are particularly vulnerable and marginalized because laws, policies and practices do not protect them from discrimination and exclusion (e.g. LGBTI people). |
| --- |
| Source: United Nations. (2020) A UN framework for the immediate socioeconomic response to COVID-19. New York, NY: United Nations. |

- *Please fill out this form*

| **Vulnerable population** | **Reason for selecting this population^*^ and current health policies for them (or policy recommendation)** |
| --- | --- |
| 1st priority: |  |
| 2nd priority: |  |
| 3rd priority: |  |
| Marginalized and vulnerable group not listed above |  |
| ^*^ If there was a special event, please describe the details such as date and situation. Please insert references or materials if possible. | |

1. Please describe and rate the indicators below focused on the marginalized and vulnerable populations during the COVID-19 pandemic in your country.

- *These indicators are based on Global Health Security Index. Please refer recent GHS methodology report and raw data files for the descriptions of the indicators and country results (*[*https://www.ghsindex.org/report-model/*](https://www.ghsindex.org/report-model/)*).*

| **Category** | **Indicator** | **Description** | **Health policy for the marginalized and vulnerable pops** | |
| --- | --- | --- | --- | --- |
|  |  |  | **Describe specific health policies** | **Rate strength of policy and implementation** |
| Prevention | Testing | Target people, Accessibility, Fee | *E.g., Anonymous free test for everyone who visits designated public health care center*  *-Target people: …*  *-Accessibility: …*  *-Fee: …*  *-Reference: URL* | *5-point Likert scale*  *0: none*  *1: very unsatisfied*  *2: unsatisfied*  *3: satisfied*  *4: very satisfied* |
|  | Vaccine | Target people, Accessibility, Fee |  |  |
| Detection | Surveillance  (GHS 2.4.4) | Ethical considerations during surveillance |  |  |
|  | Case-based investigation  (GHS 2.5.1) | Case investigation and contact tracing, quarantine, isolation |  |  |
| Response | Emergency preparedness and response planning  (GHS 3.1.1c) | If an overarching national public health emergency response plan is in place, does it include considerations for pediatric and/ or other vulnerable populations? |  |  |
|  | Risk communication  (GHS 3.5.1b) | Does the risk communication plan outline how messages will reach populations and sectors with different communications needs (e.g., different languages, within the country, media reach)? |  |  |
|  | Access to communications infrastructure  (GHS 3.6.1~3.6.4) | Access to a mobile phone or internet |  |  |
| Health system | Health capacity in clinics, hospitals, and community care centers  (GHS 4.1.1~4.1.2) | Available human resources for the broader healthcare system, facilities capacity |  |  |
|  | Healthcare access  (GHS 4.4.1~4.4.2) *except 4.4.1c | Access to healthcare, guaranteed paid sick leave |  |  |
|  | Coverage health insurance  (GHS 4.4.1c) | Medicare/Medicaid, out of pocket expenditures |  |  |

1. Please provide the COVID-19 health outcomes in vulnerable populations.

*※ If there are no specific statistics, an expert opinion can be substituted.*

| **Category** | **COVID-19 Morbidity/Mortality** |
| --- | --- |
| Health outcome | **1) The number of confirmed COVID-19 cases and deaths**   \| First confirmed case of COVID-19: \| *First date of data* \| \| --- \| --- \| \| Index date: \| *Last date of data* \|  \|  \| Population ^1)^ \| Cases ^2)^ \| Death ^3)^ \| \| --- \| --- \| --- \| --- \| \| Total \|  \|  \|  \| \| Gender \|  \|  \|  \| \| Men \|  \|  \|  \| \| Women \|  \|  \|  \| \| Age \|  \|  \|  \| \| 80+ \|  \|  \|  \| \| 70-79 \|  \|  \|  \| \| 60-69 \|  \|  \|  \| \| 50-59 \|  \|  \|  \| \| 40-49 \|  \|  \|  \| \| 30-39 \|  \|  \|  \| \| 20-29 \|  \|  \|  \| \| 10-19 \|  \|  \|  \| \| 0-9 \|  \|  \|  \| \| 1) Please write the source of population data (URL)  2) Cumulative confirmed COVID-19 cases (please write the data source)  3) Cumulative deaths among confirmed COVID-19 cases (please write the data source) \| \| \| \|   **2) Statistics for other vulnerable population**  *Please describe the confirmed COVID-19 cases and deaths by subgroup, if applicable.* |

1. Please evaluate the change of the indicators below, focused on the marginalized and vulnerable populations during the COVID-19 pandemic in your country.

- *These indicators are based on Global Health Security Index. Please refer recent GHS methodology report and raw data files for the descriptions of the indicators and country results (https://www.ghsindex.org/report-model/).*
- *The opinion of the researchers does not have to be consistent with the GHS results. Please write the researchers’ opinion on the indicator.*

| **Category** | **Indicator** | **Description** | Change level before the COVID-19 pandemic in general population | Change level before the COVID-19 pandemic in vulnerable populations |
| --- | --- | --- | --- | --- |
| Social resilience | Literacy | literacy rate | *From -5 to +5*  *-5: extremely negative change*  *-1: slightly negative change*  *0: same as before*  *+1: slightly positive change*  *+5: extremely positive change* | *From -5 to +5*  *-5: extremely negative change*  *-1: slightly negative change*  *0: same as before*  *+1: slightly positive change*  *+5: extremely positive change* |
|  | Poverty | Economic polarization, poverty rate, etc. |  |  |
|  | Employment | Employment in the informal sector, unemployment, etc. |  |  |
|  | Coverage social insurance | Social security, unemployment insurance etc. |  |  |
|  | Public confidence in government | Policy trust and compliance in the field |  |  |
|  | Local media and reporting | media coverage robustness, open and free discussion of public issues |  |  |
|  | Inequality | Social, economic, health, etc. |  |  |
| Public health vulnerability | Access to quality healthcare excluding COVID-19 | The change of life expectancy, non-communicable mortality rate |  |  |
|  | Public healthcare spending levels per capita excluding COVID-19 |  |  |  |

1. Please answer the questions below. This information will be used to identify real-world problems and health issues in the marginalized and vulnerable populations in your country during the COVID-19 pandemic.
2. Please recommend representative newspapers (at least 3) in your country.

|  |
| --- |

1. If there are other sources that can help understand the real-world problems in those populations, please send us website links or materials.

|  |
| --- |
